# Detection and molecular characterization of two canine circovirus genotypes co-circulating in Vietnam

**DOI:** 10.1080/01652176.2021.1967511

**Published:** 2021-08-24

**Authors:** Nguyen Manh Tuong, Chutchai Piewbang, Anudep Rungsipipat, Somporn Techangamsuwan

**Affiliations:** a International Graduate Program in Veterinary Science and Technology (VST), Faculty of Veterinary Science, Chulalongkorn University, Bangkok, Thailand; b Faculty of Veterinary Medicine, Vietnam National University of Agriculture, Hanoi, Vietnam;; c Animal Virome and Diagnostic Development Research Group, Faculty of Veterinary Science, Chulalongkorn University, Bangkok, Thailand; d Department of Pathology, Faculty of Veterinary Science, Chulalongkorn University, Bangkok, Thailand

**Keywords:** canine, dog, canine circovirus, genotype, Vietnam

## Abstract

**Background:**

Canine circovirus is reported in dogs in many countries, including the USA, China and Thailand. It has been detected in healthy dogs and dogs with diarrhea, hemorrhagic gastroenteritis, and vasculitis. It comprises five genotypes and is frequently found as a coinfection with canine parvovirus-2 (CPV-2).

**Aim:**

To characterize canine circovirus genotypes co-circulating with CPV-2 in Vietnam.

**Method:**

PCR assessment of 81 CPV-2-positive fecal samples from Vietnamese diarrheic dogs up to seven months of age for other viral enteric pathogens, including canine bocavirus, canine adenovirus, paramyxovirus, canine coronavirus, porcine circovirus-3 and canine circovirus. In addition, eight selected full genome sequences of Vietnamese canine circovirus were analyzed and used for phylogeny.

**Results:**

In total 19.8% of samples were found to be positive for canine circovirus. Phylogeny revealed that the Vietnamese canine circovirus strains were clustered in two different genotypes (genotype-1 and -3). The genetic diversity among Vietnamese canine circovirus was 86.0–87.2%. The nucleotide discrepancy among both genotypes altered the deduced amino acid sequence in 14 and ten residues of the replicase and capsid proteins, respectively. Genetic recombination analysis revealed that the Vietnamese canine circovirus-6 strain has the American and Chinese canine circovirus as its major and minor parents, respectively. Only a single dog revealed triple detections of CPV-2c, Canine circovirus and canine adenovirus (1.2%).

**Conclusion:**

The co-circulation of two different genotypes of canine circovirus and CPV-2c in dogs in Vietnam has been illustrated.

**Clinical relevance:**

The mortality rate with CPV-2 only (22%) doubled in dogs with canine circovirus and CPV-2 co-infection.

## 1. Introduction

Canine circovirus (CanineCV) is a member of the genus *Circovirus*, family *Circoviridae*, along with other mammal and avian circoviruses that have been found in humans, pigs, ducks, geese and pigeons (Walker et al. 2020). In non-human mammals, the porcine circovirus (PCV) infection in pigs causes an immunosuppressive condition, particularly PCV type 2 (PCV-2), and results in devastating syndromes for the swine industry (Segales et al. 2005). The CanineCV was first detected in serum samples collected from dogs in the USA (Kapoor et al. 2012). Subsequently, the virus has been reported in dogs in Italy, Germany, Thailand, Taiwan, Argentina, China, Brazil, and Colombia (Li et al. 2013; Decaro et al. 2014; Hsu et al. 2016; Weber et al. 2018; Piewbang et al. 2018b; Kotsias et al. 2019; Sun et al. 2019; Giraldo-Ramirez et al. 2020). CanineCV is considered to be the second, non-human mammalian circovirus. Recently, CanineCV has been divided into five genotypes (CanineCV-1, -2, -3, -4, and -5) (Urbani et al., 2021). The CanineCV-1 genotype has been identified in dogs, wolves and a badger from the USA, Europe and China, which corresponds to the Cosmopolitan genotype proposed by Giraldo-Ramirez et al. (2020). The CanineCV-2, -3, and -4 genotypes are reported in Asia, including China and Thailand (Niu et al. 2020; Urbani et al., 2021), and this classification agrees with the China, Asia-I, and Asia-II genotypes reported by Giraldo-Ramirez et al. (2020). Additionally, the CanineCV-5 genotype has been detected only in the arctic foxes and red foxes in Norway (Urbani et al., 2021).

CanineCV is an icosahedral, non-enveloped virus containing a small monomeric single circular strand DNA of 2,063 nucleotides in length. Its genome is comprised of two major putative open reading frames (ORFs) that encode for replicase (Rep) and capsid (Cap) protein. The Rep protein is comprised of 303 amino acids and is essential for viral replication (Cheung 2003), while the Cap protein is comprised of 270 amino acids and plays the role of a structural protein in the virus (Kapoor et al. 2012; Li et al. 2013). Among the two major ORFs, there is a non-coding region containing nine nucleotides (TAGTATTAC), which is a thermodynamically stable stem-loop that acts during the initiation of rolling-circle replication (Kapoor et al. 2012). Recently, an additional ORF, ORF-3, was identified in the antisense of ORF1 of a Thai strain of CanineCV, but its function has yet to be elucidated (Piewbang et al. 2018b).

CanineCV has been detected in dogs with various pathological conditions, including vasculitis, gastroenteritis, and infectious respiratory disease complex (Li et al. 2013; Decaro et al. 2014; Anderson et al. 2017; Piewbang et al. 2018b). Since viral isolation *in vitro* has not been successful, knowledge on the pathogenesis of CanineCV infection in dogs remains poorly understood. However, CanineCV has been considered as a cause of enteritis and is frequently identified in dual infections attributed to canine parvovirus-2 (CPV-2) (Hsu et al. 2016; Thaiwong et al. 2016; Giraldo-Ramirez et al. 2020), leading to an increased mortality rate (Anderson et al. 2017). Therefore, it has been suggested that CanineCV likely acts in synergy with other infectious agents in the development of disease. A few studies in dogs have reported coinfections of CanineCV with CPV-2 and that these coinfected dogs developed disease with severe clinical signs (Thaiwong et al. 2016; Kotsias et al. 2019).

In Vietnam, outbreaks of CPV-2 occur frequently and cause massive numbers of deaths in dogs, which may be caused by the humid subtropical climate and poor vaccination programs. However, the study of CanineCV in Vietnam is limited. This study, therefore, investigated the incidence of CanineCV in CPV-2-infected dogs and characterized the molecular genetics of Vietnamese CanineCV (CanineCV-VN) strains using phylogenetic analysis and recombination analysis.

## 2. Materials and methods

### 2.1. Samples, viral nucleic acid extraction and CPV-2 detection

Eighty-one diarrheic dogs were presented to veterinary hospitals for medical treatment. Fecal swabs were collected from all dogs exhibiting CPV-positive detection upon immunosorbent assay (Nguyen Manh et al. 2021). Tested dogs resided in Hanoi (n = 41), Da Nang (n = 16), and Ho Chi Minh (n = 24), Vietnam, during September-December 2017. The swabs were immersed in sterile phosphate buffer saline (pH 7.4) and preserved at −80 °C until assayed. All procedures were approved by the Chulalongkorn University Animal Care and Use Committee (No. 2031005). Samples were subjected to viral nucleic acid extraction using a commercial extraction kit (Geneaid Biotech, New Taipei city, Taiwan) and further assayed via polymerase chain reaction (PCR) to confirm the presence of CPV-2, as previously described (Nguyen Manh et al. 2021). These positive amplicons were then subjected to genomic sequencing to elucidate the original genotype of the obtained CPV-2. Essential data on the animals, including their sex, age, breed, vaccination history, and clinical outcome were also documented (Supplementary Table S1).

**Table 1.** Detection of other enteric viruses as a coinfection with canine parvovirus-2 (CPV-2) in 81 Vietnamese dogs.

Type of infection	**Viruses** ^a^	Positive samples	Vaccination for CPV-2			Clinical outcome		
			Yes	No	**NA** ^b^	Death	Alive	**NA** ^b^
Single infection	CPV-2	54 (66.6%)	37	13	4	12	42	0
Dual infection	CanineCV	16^c^ (19.8%)	15	1	0	7	8	1
	CBoV	5 (6.2%)	3	2	0	1	4	0
	CAdV	3 (3.7%)	3	0	0	0	2	1
	PMX	2 (2.5%)	2	0	0	0	2	0
	CCoV	0	0	0	0	0	0	0
	PCV-3	0	0	0	0	0	0	0
Triple infection	CanineCV and CAdV	1 (1.2%)	1	0	0	0	1	0

^a^ CanineCV: Canine circovirus, CBoV: Canine bocavirus, CAdV: Canine adenovirus, PMX: Paramyxovirus, CCoV: Canine coronavirus, and PCV-3: Porcine circovirus-3.

^b^ ^ ^NA: no data available.

^c^ Number of positive samples: Hanoi (n = 10), Ho Chi Minh (n = 4), and Da Nang (n = 2).

### 2.2. PCR amplification and full-length genome sequencing of CanineCV

The primer pairs, including CanineCV-605F/-1041R and CanineCV-1022F/-1538R, were retrieved from a previous study (Piewbang et al. 2018b), while CanineCV-1448F/-110R and CanineCV-2014F/-776R were designed based on the alignment of the available CanineCV sequences from the GenBank database. These were used to detect and amplify the complete genome of the CanineCV strains obtained in this study (Supplementary Table S2). The PCR was performed using Gotaq Green Master mix (Promega, USA) and amplified the target amplicons according to the specific primer pairs. The thermal cycling was performed at an initial temperature of 94 °C for 7 min, followed by 35 cycles of 94 °C for 30 sec, 55 °C for 5 min and 72 °C for 1 min, and then a final 72 °C for 7 min. The PCR products were resolved on a 1.0% (w/v) agarose gel containing 0.5% (v/v) ethidium bromide in-gel staining and visualized under a UV transilluminator. All PCR products were purified using NucleoSpin Extract II (Macherey-Nagel, Düren, Germany) and submitted for bi-directional Sanger’s sequencing (Macrogen, Seoul, Korea), to confirm the specificity. Selected positive samples were further characterized and sequenced to obtain the full-length genome for subsequent analysis.

**Table 2.** Nucleotide composition of Vietnamese Canine circovirus (CanineCV-VN) strains.

Strain	Accession no	Nucleotide composition (2,063 nt)	
		G + C (%)	A + T (%)
UCD1-1698^a^	NC_020904	52.00	48.00
CanineCV-VN-1	MT740194	52.35	47.65
CanineCV-VN-2	MT740195	52.21	47.79
CanineCV-VN-3	MT740198	52.21	47.79
CanineCV-VN-4	MT740201	52.16	47.84
CanineCV-VN-5	MT740199	51.91	48.07
CanineCV-VN-6	MT740196	52.40	47.60
CanineCV-VN-7	MT740197	51.62	48.38
CanineCV-VN-8	MT740200	52.25	47.75

a Reference strain of CanineCV.

Other canine viral enteric pathogens, including canine bocavirus (CBoV), canine adenovirus (CAdV), paramyxovirus (PMX), canine coronavirus (CCoV) and porcine circovirus-3 (PCV-3), previously reported circovirus in dogs, were also identified using specific primers and PCR programs, as previously reported (Posuwan et al. 2010; Piewbang et al. 2017; Zhang et al. 2018; Piewbang et al., 2018a).

### 2.3. Genetic characterization and phylogenetic and amino acid analyses

The derived full-length genome sequences were aligned using the Multiple Alignment algorithm in the Fast Fourier Transform (MAFFT) program version 7 (https://mafft.cbrc.jp). They were then compared to the reference CanineCV sequence (NC_020904) and those CanineCV sequences available in the GenBank database. These alignments were then further used for nucleotide and deduced amino acid sequence analysis using the BioEdit software package version 7.2 (http://www.mbio.ncsu.edu). The full-length genome sequences and individual *Rep* and *Cap* gene sequences of the CanineCV-VN strains of this study were then subjected to further genetic analyses in order to construct maximum likelihood phylogenetic trees. A dataset of 72 full-genome CanineCV sequences, originally derived from domestic dogs, wolves, foxes and a badger between 1996 and 2020 and available in the GenBank were used to accomplish this analysis. The best-fit model of substitution (TN93 + I + G) was determined using a program implemented in the MEGA 7.0 software (https://www.megasoftware.net/). Phylogenies were constructed via maximum likelihood (ML) methods with the bootstrapping (BS) of 1,000 replicates and accepting significance at BS values > 70%. Prediction of potential N-linked glycosylation was performed using the NetNglyc 1.0 (http://www.cbs.dtu.dk/services/NetNGlyc/) (Gupta and Brunak, 2002).

### 2.4. Recombination analysis

Recombination analysis was implemented using two independent programs, The Recombination Detection Program (RDP) and Simplot analysis, to define the potential natural genetic recombination events in the evolution of the CanineCV-VNs. Initially, the possibility of genetic recombination in CanineCV-VN was statistically analyzed using GENECONV, BootScan, MaxChi, Chimaera, SiScan and 3Seq with the default settings for all parameters, which were embedded in RDP package version 4.0 (http://web.cbio.uct.ac.za/). A potential positive recombination was recorded when at least four of the six methods showed a breakpoint signal with *p*-values of < 0.01. The recombinant breakpoint was then further confirmed using bootscanning and a similarity plot, implanted in the Simplot software package v. Beta 4.94 (Piewbang et al. 2018b).

## 3. Results

### 3.1. Detection of CanineCV in CPV-2-positive Vietnamese dogs

Among 81 tested samples, 80 (98.8%) samples were found to be positive for CPV-2c, while a single sample (1.2%) was positive for CPV-2a (Supplementary Table S1). All 81 CPV-2-infected samples were then examined for CanineCV genomic detection, revealing 16 positive samples (19.8%) via the presence of the target PCR amplicons of CanineCV (Table 1). Of note, CanineCV was identified in the fecal samples of all dogs positive for the CPV-2c genotype. The rate of CanineCV detection was highest in the samples from Hanoi (24.4%, 10/41), followed by Ho Chi Minh (16.7%, 4/24), and Da Nang (12.5%, 2/16). Other canine pathogens were simultaneously detected, including CBoV (6.2%, 5/81), CAdV (3.7%, 3/81), and PMX (2.5%, 2/81). However, there were no detected cases of CCoV and PCV-3 coinfection in all the tested dogs (Table 1).

Dogs in this study that showed coinfection with CanineCV and CPV-2c were purebred (five Poodles, three Pomeranians, two Pugs, two Chihuahuas, two Bulldogs, one Malinois, and one Shih Tzu). The dogs’ ages ranged from 2 to 7 months old, with the highest prevalence at 3 months (37.5%, 6/16). Of note, only one dog (No.2 from Hanoi) revealed a triple detection of CPV-2c, CanineCV and CAdV (1.2%). The clinical signs of all dogs varied from mild to severe, including fever, depression, anorexia, vomiting, and diarrhea (data not shown). Sixty-one (75.3%) dogs were vaccinated for CPV-2, while other 41 (19.8%) and four (4.9%) dogs were unvaccinated or had no data available, respectively. The clinical outcome revealed whether dogs were dead or alive. The mortality rate was shown to be 22.2% (12/54) when dogs were infected with only CPV-2, while this percentage was double in dogs with CanineCV and CPV-2 co-infection (43.8%, 7/16) (Table 1 and Supplementary Table S1).

### 3.2. Genome organization and phylogenetic analysis of Vietnamese CanineCV

Eight out of 16 CanineCV-positive samples were further PCR amplified and sequenced using the respective primers (Supplementary Table S2) to obtain the full-length genome sequence of CanineCV. The obtained genome sequences of the CanineCV-VN strains were submitted to GenBank (MT740194-MT740201). A sequence analysis of these CanineCV-VN strains revealed a mean nucleotide composition of 52.14 ± 0.26% GC and 47.86 ± 0.25% AT, which was similar to the nucleotide composition of the reference CanineCV UCD1-1698 strain (NC_020904) from the USA (Table 2). The *Rep* gene located at nt 1-912 and encoded for 304 amino acids, while the *Cap* gene was placed in the antisense nucleotide sequences from nt 1,116–1,928 and encoded for 271 amino acids. The thermodynamical stem-loop sequence (TAGTATTAC) was located at nt 2,012–2,020.

**Figure 1.**
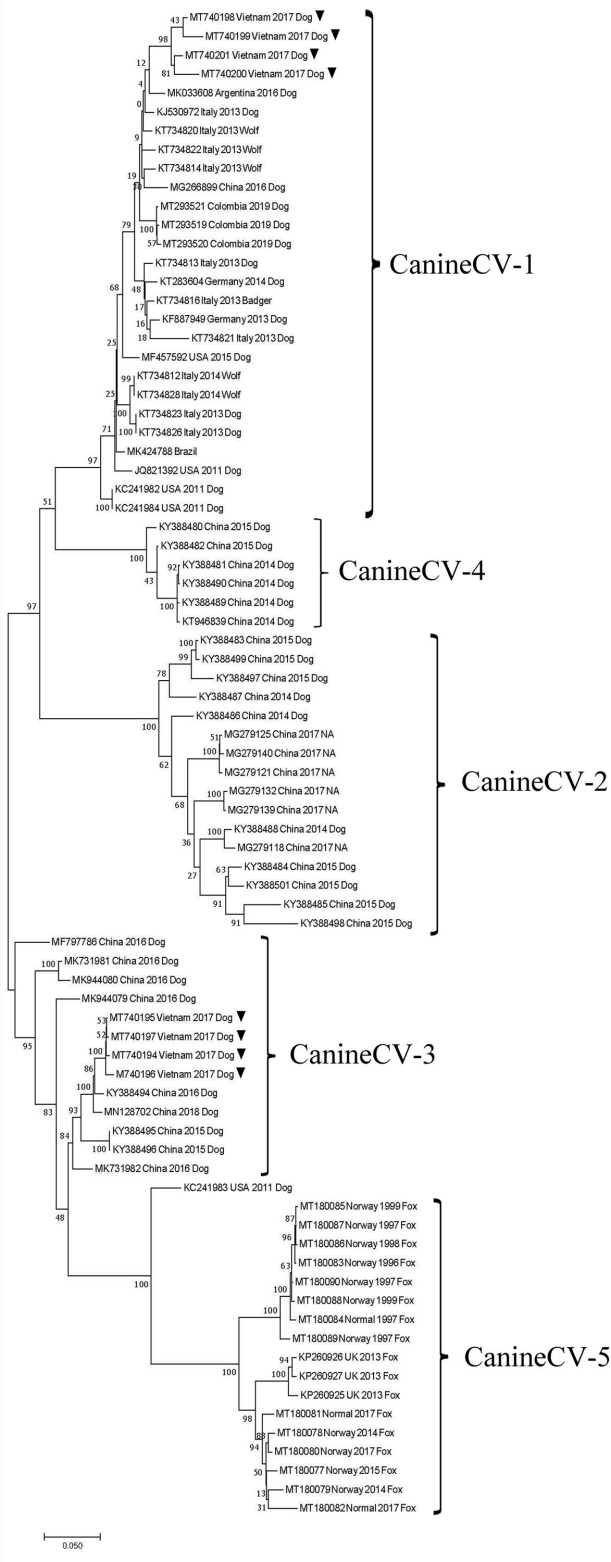
Phylogenetic tree (ML) with time, based on 80 full-length genomes of Canine circovirus (CanineCV) collected during 1996 to 2020 (▼ CanineCV-VN strains in this study).

The ML phylogenetic trees were constructed based on the eight full-genome CanineCV-VN sequences derived from this study and 72 sequences published from 1996 to 2020 and retrieved from GenBank. A total of 80 CanineCV strains were segregated into five clades, corresponding with five genotypes (Urbani et al., 2021). The CanineCV-VN-3,- 4, -5, and -8 strains were clustered together with the CanineCV-1 genotype strain, which was comprised of strains from Italy, Germany, Argentina, Colombia, Brazil, the USA and China. In contrast, the CanineCV-VN-1, -2, -6, and -7 strains were grouped in the CanineCV-3 genotype, along with other strains from China (Figure 1). The phylogenetic analyses based on the individual *Rep* and *Cap* genes also showed similar ML phylogenetic trees results as compared to the phylogeny based on the full genomes (Supplementary Figures S1 and S2).

**Figure 2.**
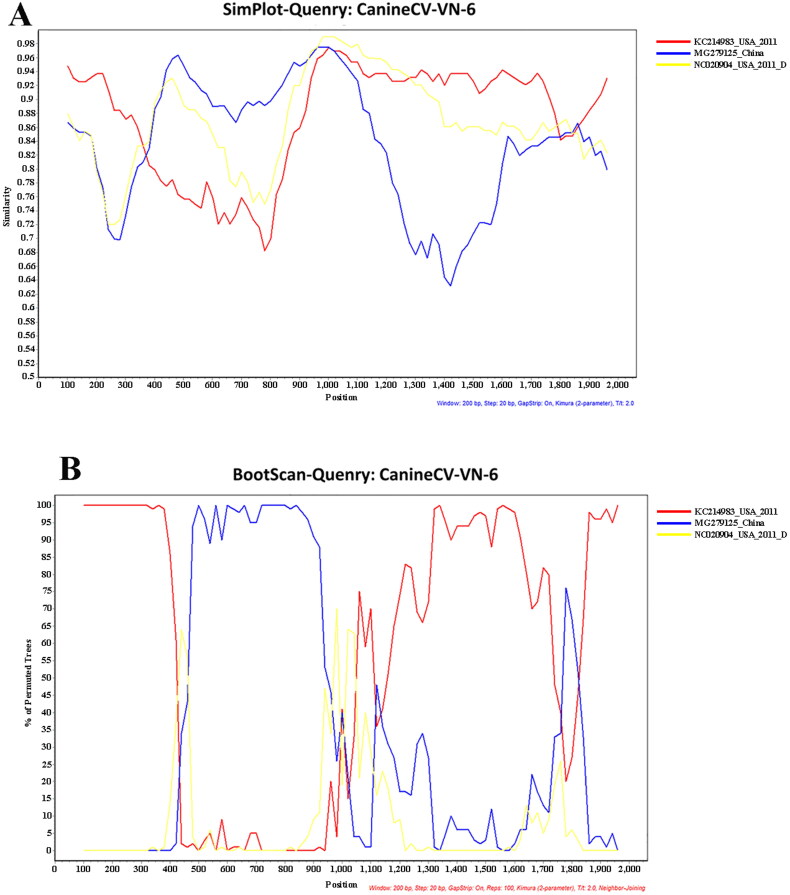
Potential recombination events of Vietnamese Canine circovirus (CanineCV-VN-6 as query) strains compared to published CanineCV strains; (A) Similarity plot, and (B) Bootscan analysis.

### 3.3. Genetic and amino acid characterizations of different CanineCV genotypes in Vietnam

A genetic analysis of eight full-length genomes revealed that CanineCV-VN strains shared 86.0–99.8% similarity. Meanwhile, the nucleotide similarities among strains of the Vietnamese CanineCV-1 genotype (CanineCV-VN-3, -4, -5, and -8) and Vietnamese CanineCV-3 genotype (CanineCV-VN-1, -2, -6, and -7) were 95.2–98.0% and 98.9–99.8%, respectively. However, the nucleotide comparison between both Vietnamese genotypes was 86.0–87.2% (Table 3 and Supplementary Table S3a). In addition, the nucleotide similarity analysis among genotypes revealed 83.8–85.9% and 85.3–86.8% similarity for the *Rep* and *Cap* genes, respectively. The deduced amino acid similarities between the Vietnamese CanineCV-1 and CanineCV-3 genotypes were 92.7–97.3% for the Rep protein and 92.9–95.1% for the Cap protein (Table 3 and Supplementary Table S3b and S3c).

**Table 3.** Similarity percentage of nucleotides and deduced amino acids of Vietnamese Canine circovirus (CanineCV-VN) and reference strains.

Genotype	Strain	Sequence	Similarity (%)		
			Full genome	Replicase	Capsid
	UCD1-1698^a^ & CanineCV-VN-3, 4, 5, 8	Nucleotide Amino acid	93.6–94.4	94.7–96.7 95.7–97.3	91.3–92.9 97.0–97.7
	UCD1-1698^a^ & CanineCV-VN-1, 2, 6, 7	Nucleotide Amino acid	88.3–88.5	85.7–86.1 93.7–94.3	89.1–89.6 98.8–95.9
CanineCV-1	CanineCV-VN-3, 4, 5, 8	Nucleotide Amino acid	95.2–98.0	95.3–98.6 96.0–99.3	94.2–99.0 97.0–99.6
CanineCV-3	CanineCV-VN-1, 2, 6, 7	Nucleotide Amino acid	98.9–99.8	98.4–99.8 93.7–100.0	99.0–99.8 98.5–100.0
CanineCV-1 & -3b		Nucleotide Amino acid	86.0–87.2	83.8–85.9 92.7–97.3	85.3–86.8 92.9–97.7

^a^ Reference strain of CanineCV (Accession no. NC_020904); similarities were compared between UCD1-1698 strains and CanineCV-VN strains.

^b^ Similarities were compared between Vietnamese CanineCV-1 and -3 genotypes.

Between the Vietnamese CanineCV-1 and CanineCV-3 genotypes, amino acid variations were clearly observed in 14 amino acid positions of the Rep protein and ten amino acid positions of the Cap protein (Table 4). Among these positions, almost all the amino acid sequences of the Rep and Cap proteins of the Vietnamese CanineCV-1 genotype were identical to the reference UCD1-1698 strain (NC_020904), except for amino acids at 269 (Rep) and 74 (Cap). A further analysis illustrated the unique amino acid variations in both the Rep and Cap proteins of each CanineCV genotype, as shown in Table 5 and Supplementary Tables S4 and S5. In addition, a single potential N-glycosylation site at position N134AT was found in the Cap protein of seven of the CanineCV-VN strains, but only one strain (CanineCV-VN-7) presented H134AT. However, no N-glycosylation site was found in the Rep protein.

**Table 4.** Amino acid^a^ variations in the Replicase (Rep) and Capsid (Cap) proteins of Vietnamese canine circovirus (CanineCV-VN) strains.

**Amino acid sequence of Rep protein** ^b^																																				
	10	**56**		**69**	**71**		78	**97**		106	**110**		**113**		115	126		131	**140**		**149**		168	174		194		**248**	**252**		**265**		**268**	**269**		**288 (aa)**
NC_020904^c^	G	N		R	T		A	R		L	S		P		S	V		T	S		Y		N	E		E		L	I		S		I	G		N
CanineCV-VN-1	V	S		K	C		A	K		I	A		V		R	V		T	A		F		T	E		S		V	V		N		F	G		S
CanineCV-VN-2	V	S		K	C		A	K		I	A		V		R	V		T	A		F		T	E		S		V	V		N		F	G		S
CanineCV-VN-6	V	S		K	C		A	K		I	A		V		R	V		T	A		F		T	E		S		V	V		N		F	G		S
CanineCV-VN-7	V	S		K	C		A	K		I	A		V		R	V		T	A		F		T	E		S		V	V		N		F	G		S
CanineCV-VN-3	V	N		R	T		G	R		I	S		P		R	I		S	S		Y		T	Q		E		L	I		S		V	P		N
CanineCV-VN-4	V	N		R	T		G	R		I	S		P		R	I		S	S		Y		T	Q		E		L	I		S		V	P		N
CanineCV-VN-5	G	N		R	T		A	R		I	S		P		S	V		T	S		Y		K	E		D		L	I		S		V	P		N
CanineCV-VN-8	V	N		R	T		G	R		I	S		P		R	I		S	S		Y		T	Q		E		L	I		S		V	P		N

**Amino acid sequence of Cap protein** ^b^																																				
	**13**		14			**16**			**29**			**58**		**74**			**101**			**111**		148			**149**		195			211		**240**			**242 (aa)**	
NC_020904^c^	S		Y			T			R			T		T			Y			K		R			L		A			V		D			S	
CanineCV-VN-1	R		F			A			N			Q		T			F			R		I			G		T			V		E			T	
CanineCV-VN-2	R		F			A			N			Q		T			F			R		I			G		T			V		E			T	
CanineCV-VN-6	R		F			A			N			Q		T			F			R		I			G		T			V		E			T	
CanineCV-VN-7	R		F			A			N			Q		T			F			R		I			G		T			V		E			T	
CanineCV-VN-3	S		Y			T			R			T		S			Y			K		R			L		T			I		D			S	
CanineCV-VN-4	S		F			T			R			T		S			Y			K		T			L		T			I		D			S	
CanineCV-VN-5	S		Y			T			R			T		S			Y			K		T			L		T			I		D			S	
CanineCV-VN-8	S		F			T			R			T		S			Y			K		R			L		T			V		D			S	

^a^ Amino acid symbol: A (Alanine), R (Arginine), N (Asparagine); D (Aspartic acid), Q (Glutamine), G (Glycine), I (Isoleucine), L (Leucine), K (Lysine), M (Methionine), F (Phenylalanine), P (Proline), S (Serine), and T (Threonine).

^b^ Bold numbers indicate the distinct amino acid variations between Vietnamese CanineCV-1 and -3 genotypes.

^c^ Reference strain.

**Table 5.** Relevant amino acid^a^ changes in the Replicase (Rep) and Capsid (Cap) protein sequence in each Canine circovirus (CanineCV) genotypes.

	Rep protein																										
Genotype	32		35			69			97		141		149			164		177		211			231		249		
CanineCV-1	E		D			R/Q			R		Y/F		Y/F			T/A		C		Q			C/T		A		
CanineCV-2	A/E		D/E			N/K			R		Y		F/Y			T/A/N		C		Q/P/S			L		A		
CanineCV-3	E		D/E			K			K		Y		F			T		C		Q			L/C		A		
CanineCV-4	E		E			N			R		Y/F		Y			T		C		Q			L		A		
CanineCV-5	E		D			K/R			R/Q		H		H			A/T		V		S			C		G		
		
	**Cap protein**																										
**Genotype**	**13**	**28**		**29**	**57**		**83**	**94**		**95**		**144**		**149**	**150**		**193**		**195**		**208**	**211**		**239**		**240**	
CanineCV-1	S/N	N		R	Q		T/I	F/Y		Y		T/S/C		L	E		D/N		T/A		T	V/I		P		D	
CanineCV-2	R	N		N	T/Q		T	Y		F		T/S		S/N	Q		E		Q		N/T	I		A		E	
CanineCV-3	R/S	N		N/T	Q		T/A	F/Y		Y/F/H		T		G/H/S	E		D		T		T	V		P		E	
CanineCV-4	R	N		N	Q		T	Y/F		Y		T		M	E		D		T		T	I		P		D	
CanineCV-5	R	R		N	Q/R		V	Y		F/Y		A/T		T/N/K	E/D		E/D		V/A/S/T		Q	V		P/L/S		E/D	

^a^ Amino acid symbol: A (Alanine), R (Arginine), N (Asparagine); D (Aspartic acid), C (Cysteine), E (Glutamic acid), Q (Glutamine), G (Glycine), I (Isoleucine), L (Leucine), K (Lysine), M (Methionine), F (Phenylalanine), P (Proline), S (Serine), T (Threonine), Y (Tyrosine), and V (Valine)

### 3.4. Recombination analysis

Recombination analysis was applied to all eight CanineCV-VN strains obtained in this study using RDP4 software, including RDP, GENECONV, Bootscan, Maxchi, Chimaera, SiScan, 3Seg and LARD. A potential recombination breakpoint was found in the Vietnamese CanineCV-1 genotype (CanineCV-VN-3, -4, -5, and -8). However, the potential recombination signal was only confirmed in the CanineCV-VN-6 strain (Vietnamese CanineCV-3 genotype), located at nucleotide position 420–1020. The recombinant CanineCV-VN-6 strain had an American CanineCV (KC241983, as an outgroup strain) and a Chinese CanineCV (MG279125, CanineCV-2 genotype) as its major and minor parents, respectively (Figure 2).

## 4. Discussion

This study reports the first identification of CanineCV in Vietnam and provides evidence of CanineCV circulation in Vietnamese dogs. The CanineCV genome was detected in CPV-2-positive fecal samples at an incidence of 19.8**%,** which is higher than those reported in Germany **(**13.0**%)** and South America **(**16.6**%)** (Anderson et al. 2017; Giraldo-Ramirez et al. 2020). The incidence of CanineCV detected from this study varied in the Northern, Central and Southern areas of Vietnam. The fecal samples collected in Hanoi (Northern Vietnam) showed the highest rate of CanineCV at about 24.4%, while those samples from Da Nang (Central Vietnam) and Ho Chi Minh (Southern Vietnam) were about 12.5% and 16.7%, respectively. Since this study did not investigate the presence of CanineCV in either healthy or CPV-2-negative dogs and the number of samples has been limited, the actual prevalence of CanineCV in Vietnam could not be ascertained. Further investigation into these groups is required to understand the epidemiology of CanineCV in Vietnam.

The pathogenesis of CanineCV-associated disease and the potential synergistic effects of CanineCV and other pathogens have not been clarified. However, several studies have indicated that CanineCV may act in synergy with other infectious agents in the development of a disease **(**Thaiwong et al. 2016; Zaccaria et al. 2016; Kotsias et al. 2019**)**. In this study, CanineCV was co-detected with CPV-2c in enteritis-suffering dogs, which is in agreement with the previous findings (Kotsias et al. 2019; Giraldo-Ramirez et al. 2020). Since CPV-2c has been reported as a predominant genotype detected in Vietnam (Hoang et al. 2019), which is in agreement with our study, co-infection with CanineCV may play a significant role as a negative co-factor in disease outcomes in dogs with CPV-2 infection (Anderson et al. 2017). This is shown in our study as a higher mortality rate for dogs co-infected with CanineCV and CPV-2 (43.8%) than for dogs having a single CPV-2 infection (22.2%). However, a recent report indicated that the CanineCV may play a role as a primary pathogen that causes acute hemorrhagic diarrheic disease in dogs (Anderson et al. 2017). Thus, the definitive role of CanineCV-associated diseases should be explored in further studies.

Here, we described the characteristics of the full-length genomes and deduced amino acids of the eight CanineCV-VN strains in order to add information on the geographical extent of the virus. The viral genome encompasses two ORFs encoding for the Rep and Cap proteins, similar to a previous report (Kapoor et al. 2012). However, genetic diversity among the CanineCV-VN genotypes was detected. The complete genome sequences of the Vietnamese CanineCV-1 genotype showed a close similarity **(**94.4**%)** to the reference strain, but the other genotype had a lower similarity **(**88.3**%)**. The further surveillance of CanineCV genotypes is vital in understanding viral molecular genetics.

Previous studies on PCV-2 have suggested that amino acid mutations in the Rep protein may affect viral replication ability, while amino acid mutations in the Cap protein may alter viral antigenicity (Lekcharoensuk et al. 2004; Cheung 2012). Changes in several amino acids in PCV-3 are related to viral evolutionary dynamics **(**Sun et al. 2018**)**. However, knowledge of mutations in CanineCV is limited. Selective pressure analysis on the PCV-2 genome has been demonstrated to be the factor driving mutations in the *Cap* gene, resulting in the virus escaping the host’s immune response in pigs **(**Franzo et al. 2016**)**. However, the factor driving the mutation in CanineCV has not been investigated, but evidence for positive selection in both the Cap and Rep proteins of CanineCV has been reported (Giraldo-Ramirez et al. 2020). Thus, further study is still necessary to understand the evolutionary pattern of this virus.

The ML phylogenetic analysis, based on the full-length genome and the individual *Rep* and *Cap* genes, divided the CanineCV strains into five clades corresponding to five genotypes (CanineCV-1 to -5) (Giraldo-Ramirez et al. 2020). Notably, the CanineCV-1 genotype was mainly detected in Europe (Italy and Germany) and the Americas (USA, Brazil, Colombia and Argentina), except for one Chinese sequence (MG266899) (Niu et al. 2020) and four Vietnamese sequences (MT740198-MT740201) from this study. The CanineCV-2 and -4 genotypes were distributed only in China, while the CanineCV-3 genotype was detected in both China and Vietnam, representing four sequences (MT740194-MT740197) from this report. Of note, the CanineCV-5 genotype is found only in arctic foxes (*Vulpes lagopus*) and red foxes (*Vulpes vulpes*) in Norway and the UK (Bexton et al. 2015; Urbani et al., 2021). Interestingly, each genotype presented the typical mutation in both the Rep and Cap proteins. Since the geographic distribution pattern has been well-described in canine distemper virus (CDV; a fatal Morbillivirus in dogs), this indicates that the origin and global evolution of CDV derives from specific mutations of the ancestral strain (Panzera et al. 2015). Therefore, further study is required to verify this observation in CanineCV, as to whether any mutations in the ancestral sequence (KC241983 as an outgroup in the phylogeny) play a role in the global evolution of CanineCV. In addition, this study confirms that the CanineCV-1 and -3 genotypes are now circulating in CPV-2 enteritis-infected dogs in Vietnam, and provides evidence for the distribution of the CanineCV-1 genotype in Asia.

Recombination events have previously been identified in CanineCV in the *Rep* gene and other parts of the genome (Piewbang et al. 2018b; Sun et al. 2019). In this study, a recombination event was found in the CanineCV-VN-6 strain at nucleotide position 420–1020 with the major parent sequence derived from an ancestral American strain (KC241983) and a minor parent sequence derived from the Chinese strain (MG279125; CanineCV-2 genotype). In the results of the phylogenetic analysis on the *Cap* gene, we found that the ancestral American strain KC214983 has been clustered within the most CanineCV sequences detected in China and Vietnam, suggesting that they shared evolutionary patterns among sequences. This result implies that the American strain may serve as the common ancestor of the strains detected in Vietnam. Further studies on evolutionary analysis and retrospective, larger-scaled investigations in the form of genetic analysis are, therefore, needed to elucidate the intermediate CanineCV strains that may have closer relationships to the American ancestor. This viral recombination is important because it has been associated with viral host range expansion, the emergence of novel progeny viruses that express new antigenic and serological characteristic, and increases in virulence and pathogenesis (Pérez-Losada et al. 2015). Our findings suggest that various genotypes of CanineCV have been prevalent in Vietnam and a novel recombinant CanineCV-CN-6, which belonged to the CanineCV-3 genotype, is apparent. This recombination event should be strictly monitored because the different genotypes of CanineCV have simultaneously circulated and been co-detected with other pathogens in the same host.

In conclusion, this is the first detection and characterization of the full-length genome of CanineCV in Vietnam. This study provides further evidence of CanineCV as a co-infectious agent for enteritis in dogs. Phylogenetic analysis revealed that CanineCV was classified into five genotypes marked by typical mutations of amino acid and specific geographic distribution. Further studies are vital in understanding the genotypes of CanineCV.

## Supplementary Material

Supplemental Material

Supplemental Material
