# Supplementary material for: Detection and molecular characterization of two canine circovirus genotypes co-circulating in Vietnam
Source: Vet Q. 2021 Aug 24;41(1):232–41. doi: 10.1080/01652176.2021.1967511 (PMC8386738; doi:10.1080/01652176.2021.1967511)

**Supplementary figure legends**

**Supplementary Fig. S1** The phylogenetic tree (ML) with time, based on 80 sequences of the Replicase gene of Canine circovirus (CanineCV) collected during 1996 to 2020 ( indicates the Vietnamese CanineCV strains from this study).


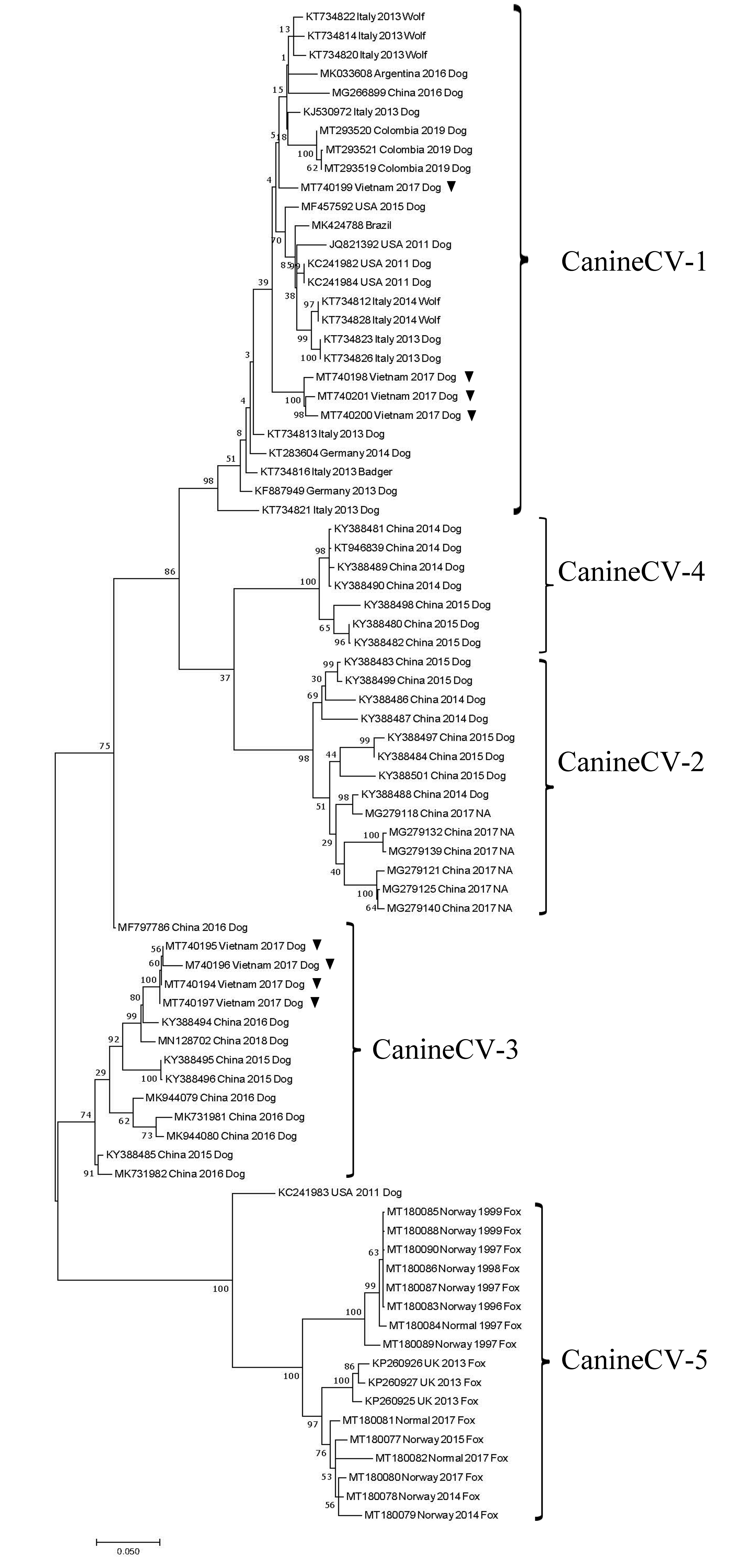


**Supplementary Fig. S2** The phylogenetic tree (ML) with time, based on 80 sequences of the Capsid gene of Canine circovirus (CanineCV) collected during 1996 to 2020 ( indicates Vietnamese CanineCV strains in this study).


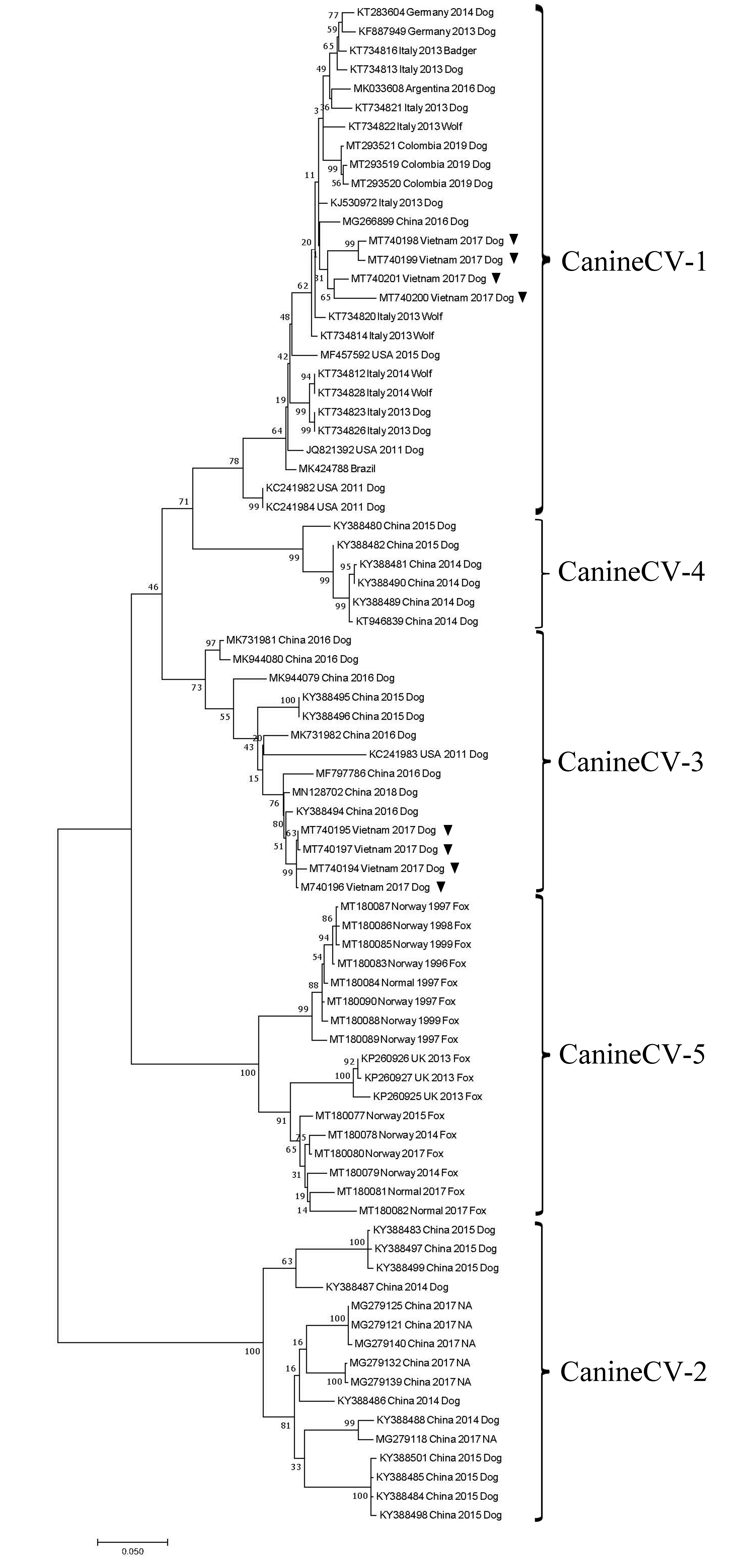

Supplement: Supplemental Material [file TVEQ_A_1967511_SM5462.doc]
