# Supplementary material for: Detection and molecular characterization of two canine circovirus genotypes co-circulating in Vietnam
Source: Vet Q. 2021 Aug 24;41(1):232–41. doi: 10.1080/01652176.2021.1967511 (PMC8386738; doi:10.1080/01652176.2021.1967511)
Supplement: Supplemental Material [file TVEQ_A_1967511_SM5446.doc]

**Supplementary Table S1** Detection of canine circovirus (CanineCV) and other pathogens from canine parvovirus-2 (CPV-2)-infected dogs in Vietnam

| **No^1^** | **Location** | **Sampling time^2^** | **Breed** | **Age (month)^3^** | **Sex^4^** | **Vaccination^5^** | **Death^3^** | **Viruses^6^** | | | | | | |
| --- | --- | --- | --- | --- | --- | --- | --- | --- | --- | --- | --- | --- | --- | --- |
|  |  |  |  |  |  |  |  | **CPV-2 Genotype** | **CanineCV** | **CBoV** | **CAdV** | **PMX** | **CCoV** | **PCV-3** |
| 1 | Hanoi | 2017/Sep | Mixed | 3 | F | N | N | 2c | - | - | - | - | - | - |
| 2^++^ | Hanoi | 2017/Sep | Poodle | 4 | F | Y | N | 2c | + | - | + | - | - | - |
| 3 | Hanoi | 2017/Sep | Bulldog | 2 | M | Y | N | 2c | - | - | - | - | - | - |
| 4 | Hanoi | 2017/Sep | Poodle | 2 | F | Y | Y | 2c | + | - | - | - | - | - |
| 5 | Hanoi | 2017/Sep | Shih Tzu | 5 | M | Y | N | 2c | - | - | - | - | - | - |
| 6 | Hanoi | 2017/Sep | Mixed | NA | F | NA | N | 2c | - | - | - | - | - | - |
| 7 | Hanoi | 2017/Sep | Mixed | 3 | F | N | N | 2c | - | - | - | - | - | - |
| 8 | Hanoi | 2017/Sep | Malinois | 4 | M | N | N | 2c | - | + | - | - | - | - |
| 9 | Hanoi | 2017/Sep | Pug | 6 | Mc | Y | Y | 2c | + | - | - | - | - | - |
| 10 | Hanoi | 2017/Sep | Poodle | 2 | F | Y | N | 2c | + | - | - | - | - | - |
| 11 | Hanoi | 2017/Sep | Poodle | 4 | M | Y | Y | 2c | - | - | - | - | - | - |
| 12 | Hanoi | 2017/Sep | Pug | 4 | M | Y | N | 2c | + | - | - | - | - | - |
| 13 | Hanoi | 2017/Sep | Pomeranian | 5 | F | NA | Y | 2c | - | - | - | - | - | - |
| 14 | Hanoi | 2017/Sep | Poodle | 3 | F | Y | Y | 2c | - | - | - | - | - | - |
| 15^++^ | Hanoi | 2017/Sep | Malinois | 2 | M | Y | N | 2c | + | - | - | - | - | - |
| 16 | Hanoi | 2017/Sep | Bulldog | 2 | M | N | N | 2c | - | - | - | - | - | - |
| 17 | Hanoi | 2017/Sep | Pug | 4 | M | Y | NA | 2c | - | - | + | - | - | - |
| 18 | Hanoi | 2017/Sep | Pug | 3 | F | Y | N | 2c | - | - | - | - | - | - |
| 19 | Hanoi | 2017/Sep | Shih Tzu | 3 | M | Y | Y | 2c | + | - | - | - | - | - |
| 20 | Hanoi | 2017/Nov | Poodle | 8 | F | Y | N | 2c | - | - | - | - | - | - |
| 21 | Hanoi | 2017/Nov | Chihuahua | 2 | F | Y | N | 2c | - | + | - | - | - | - |
| 22 | Hanoi | 2017/Nov | Pug | 5 | M | Y | N | 2c | - | - | - | - | - | - |
| 23 | Hanoi | 2017/Nov | Mixed | 2 | M | Y | Y | 2c | - | - | - | + | - | - |
| 24 | Hanoi | 2017/Nov | Mixed | 2 | M | Y | Y | 2c | - | - | - | - | - | - |
| 25 | Hanoi | 2017/Nov | Poodle | 2 | M | Y | N | 2c | - | - | - | - | - | - |
| 26 | Hanoi | 2017/Nov | Malinois | 6 | F | Y | N | 2c | - | - | - | - | - | - |
| 27 | Hanoi | 2017/Nov | Poodle | 3 | F | Y | N | 2c | - | - | - | - | - | - |
| 28^++^ | Hanoi | 2017/Nov | Pomeranian | 4 | M | Y | Y | 2c | + | - | - | - | - | - |
| 29 | Hanoi | 2017/Nov | Poodle | 3 | F | Y | N | 2c | - | - | - | - | - | - |
| 30 | Hanoi | 2017/Nov | Pomeranian | 3 | F | N | N | 2c | - | - | - | - | - | - |
| 31 | Hanoi | 2017/Nov | Dachshund | 4 | F | Y | N | 2c | - | - | - | + | - | - |
| 32 | Hanoi | 2017/Nov | Poodle | 6 | Mc | Y | N | 2c | - | - | - | - | - | - |
| 33 | Hanoi | 2017/Nov | Chihuahua | 3 | M | Y | N | 2c | + | - | - | - | - | - |
| 34 | Hanoi | 2017/Nov | Malinois | 2 | M | N | Y | 2c | - | - | - | - | - | - |
| 35 | Hanoi | 2017/Nov | Poodle | 6 | M | Y | N | 2c | - | - | - | - | - | - |
| 36 | Hanoi | 2017/Dec | Poodle | 3 | F | Y | Y | 2c | + | - | - | - | - | - |
| 37 | Hanoi | 2017/Dec | Bulldog | 4 | F | Y | N | 2c | - | - | - | - | - | - |
| 38 | Hanoi | 2017/Dec | Mixed | 3 | F | Y | N | 2c | - | - | - | - | - | - |
| 39 | Hanoi | 2017/Dec | Poodle | 3 | F | Y | N | 2c | - | - | - | - | - | - |
| 40 | Hanoi | 2017/Dec | Malinois | 4 | M | Y | N | 2c | - | - | + | - | - | - |
| 41 | Hanoi | 2017/Dec | Dachshund | 3 | M | N | N | 2c | - | - | - | - | - | - |
| 42 | Danang | 2017/Nov | Poodle | 8 | F | Y | N | 2c | - | - | - | - | - | - |
| 43 | Danang | 2017/Nov | Chihuahua | 2 | F | Y | N | 2c | - | + | - | - | - | - |
| 44 | Danang | 2017/Nov | Pug | 5 | M | Y | Y | 2c | - | - | - | - | - | - |
| 45 | Danang | 2017/Nov | Mixed | 2 | M | Y | Y | 2c | - | - | - | - | - | - |
| 46 | Danang | 2017/Nov | Mixed | 2 | M | N | Y | 2c | - | + | - | - | - | - |
| 47 | Danang | 2017/Nov | Poodle | 4 | M | N | N | 2c | - | - | - | - | - | - |
| 48 | Danang | 2017/Nov | Malinois | 3 | F | Y | N | 2c | - | - | - | - | - | - |
| 49 | Danang | 2017/Nov | Poodle | 3 | F | N | N | 2c | - | - | - | - | - | - |
| 50^++^ | Danang | 2017/Nov | Pomeranian | 4 | M | Y | Y | 2c | + | - | - | - | - | - |
| 51 | Danang | 2017/Nov | Bulldog | 2 | F | Y | N | 2c | - | - | - | - | - | - |
| 52 | Danang | 2017/Nov | Bulldog | 2 | F | Y | Y | 2c | - | - | - | - | - | - |
| 53 | Danang | 2017/Nov | Bulldog | 2 | F | Y | N | 2c | - | - | - | - | - | - |
| 54 | Danang | 2017/Nov | Pug | 6 | Mc | Y | N | 2c | - | - | - | - | - | - |
| 55^++^ | Danang | 2017/Nov | Chihuahua | 3 | M | Y | NA | 2c | + | - | - | - | - | - |
| 56 | Danang | 2017/Nov | Malinois | 4 | M | Y | N | 2c | - | - | - | - | - | - |
| 57 | Danang | 2017/Nov | Poodle | 3 | M | N | N | 2c | - | - | - | - | - | - |
| 58 | Ho Chi Minh City | 2017/Dec | Bulldog | 3 | F | Y | N | 2c | - | - | - | - | - | - |
| 59 | Ho Chi Minh City | 2017/Dec | Bulldog | 4 | F | Y | N | 2c | - | - | - | - | - | - |
| 60 | Ho Chi Minh City | 2017/Dec | Pomeranian | 4 | F | Y | N | 2c | - | - | - | - | - | - |
| 61 | Ho Chi Minh City | 2017/Dec | Pomeranian | 4 | F | Y | N | 2c | - | - | - | - | - | - |
| 62 | Ho Chi Minh City | 2017/Dec | Pomeranian | 4 | M | Y | Y | 2c | - | - | - | - | - | - |
| 63 | Ho Chi Minh City | 2017/Dec | Dachshund | 3 | M | Y | N | 2c | - | - | - | - | - | - |
| 64 | Ho Chi Minh City | 2017/Dec | Bulldog | 3 | M | N | N | 2c | - | - | - | - | - | - |
| 65^++^ | Ho Chi Minh City | 2017/Dec | Bulldog | 3 | F | N | Y | 2c | + | - | - | - | - | - |
| 66 | Ho Chi Minh City | 2017/Dec | Shih Tzu | 2 | F | Y | N | 2c | - | - | - | - | - | - |
| 67 | Ho Chi Minh City | 2017/Dec | Poodle | 3 | F | Y | N | 2c | + | - | - | - | - | - |
| 68 | Ho Chi Minh City | 2017/Dec | Bulldog | 4 | F | Y | N | 2c | - | - | - | - | - | - |
| 69 | Ho Chi Minh City | 2017/Dec | Mixed | 3 | M | N | N | 2c | - | - | - | - | - | - |
| 70 | Ho Chi Minh City | 2017/Dec | Shih Tzu | NA | M | NA | N | 2c | - | - | - | - | - | - |
| 71 | Ho Chi Minh City | 2017/Dec | Poodle | 4 | M | Y | N | 2a | - | - | - | - | - | - |
| 72^++^ | Ho Chi Minh City | 2017/Dec | Pomeranian | 7 | Fs | Y | N | 2c | + | - | - | - | - | - |
| 73 | Ho Chi Minh City | 2017/Dec | Mixed | 5 | F | Y | N | 2c | - | - | - | - | - | - |
| 74 | Ho Chi Minh City | 2017/Dec | Mixed | 5 | F | Y | N | 2c | - | + | - | - | - | - |
| 75 | Ho Chi Minh City | 2017/Dec | Bulldog | 3 | F | Y | N | 2c | - | - | - | - | - | - |
| 76 | Ho Chi Minh City | 2017/Dec | Pug | 3 | F | Y | Y | 2c | - | - | - | - | - | - |
| 77 | Ho Chi Minh City | 2017/Dec | Mixed | 2 | F | N | Y | 2c | - | - | - | - | - | - |
| 78 | Ho Chi Minh City | 2017/Dec | Poodle | 9 | Mc | N | N | 2c | - | - | - | - | - | - |
| 79 | Ho Chi Minh City | 2017/Dec | Poodle | 6 | F | NA | N | 2c | - | - | - | - | - | - |
| 80^++^ | Ho Chi Minh City | 2017/Dec | Bulldog | 2 | M | Y | N | 2c | + | - | - | - | - | - |
| 81 | Ho Chi Minh City | 2017/Dec | Bulldog | 5 | F | Y | N | 2c | - | - | - | - | - | - |

^1^ ++: Samples submitting for CanineCV sequencing

^2^ Sampling time: year/month

^3^ Y: Yes, N: No, NA: No data available

^4^ M: male, F: female, Mc: castrated male, Fs: sprayed female

^5^ Vaccination for parvovirus: Y: Yes, N: No, NA: No data available

^6^ CPV-2: Canine parvovirus type-2, CanineCV: Canine circovirus, CBoV: Canine bocavirus, CAdV: Canine adenovirus, PMX: Paramyxovirus, CCoV: Canine coronavirus, PCV-3: Porcine circovirus-3

^7^ +: positive, -: Negative

**Supplementary Table S2** Primers used in this study for the PCR-based canine circovirus (CanineCV) detection and sequencing

| **Primers** | **Sequence (5’-3’)** | **Nucleotide position** | **References** |
| --- | --- | --- | --- |
| CanineCV-605F | AATGGTGGGAYGGYTACGATGG | 605-626 | Piewbang et al., 2018b |
| CanineCV-1041R | AAGGGGGGTGAACAGGTAAAC | 1041-1021 | Piewbang et al., 2018b |
| CanineCV-1022F | TTTACCTGTTCACCCCCCTTCGA | 1022-1044 | Piewbang et al., 2018b |
| CanineCV-1538R | GGAAGAGGYAATGCTACAAGATCA | 1538-1515 | Piewbang et al., 2018b |
| CanineCV-1448F | TGAAYGGAGCCTTRTTDGGATC | 1448-1469 | This study |
| CanineCV-110R | TCCGGCGCRAGGTTCTTCA | 120-110 | This study |
| CanineCV-2014F | GTATTACCCGGCACCTCGTC | 2014-2033 | This study |
| CanineCV-776R | CATCAYTATACCAYTCATGAGGC | 798-776 | This study |

**Supplementary Table S3a** Similarity (%) of the full-genome sequences of Vietnamese Canine circovirus (CanineCV) with the NC_020904 reference strain

|  |  | **CanineCV-3** | | | | **CanineCV-1** | | | |
| --- | --- | --- | --- | --- | --- | --- | --- | --- | --- |
|  | **NC_020904** | **VN-1** | **VN-2** | **VN-6** | **VN-7** | **VN-3** | **VN-5** | **VN-8** | **VN-4** |
| **NC_020904** |  |  |  |  |  |  |  |  |  |
| **VN-1** | 0.883 |  |  |  |  |  |  |  |  |
| **VN-2** | 0.885 | 0.996 |  |  |  |  |  |  |  |
| **VN-6** | 0.883 | 0.989 | 0.992 |  |  |  |  |  |  |
| **VN-7** | 0.884 | 0.995 | 0.998 | 0.990 |  |  |  |  |  |
| **VN-3** | 0.936 | 0.862 | 0.864 | 0.863 | 0.862 |  |  |  |  |
| **VN-5** | 0.944 | 0.870 | 0.872 | 0.870 | 0.871 | 0.980 |  |  |  |
| **VN-8** | 0.941 | 0.861 | 0.863 | 0.860 | 0.862 | 0.964 | 0.952 |  |  |
| **VN-4** | 0.940 | 0.867 | 0.869 | 0.867 | 0.868 | 0.979 | 0.968 | 0.972 |  |

**Supplementary Table S3b** Similarity (%)^1^ of the Replicase gene sequences of Vietnamese Canine circovirus (CanineCV) with the NC_020904 reference strain

|  |  | **CanineCV-3** | | | | **CanineCV-1** | | | |
| --- | --- | --- | --- | --- | --- | --- | --- | --- | --- |
|  | **NC_020904** | **VN-1** | **VN-2** | **VN-6** | **VN-7** | **VN-3** | **VN-5** | **VN-8** | **VN-4** |
| **NC_020904** |  | 0.943 | 0.943 | 0.937 | 0.943 | 0.960 | 0.973 | 0.960 | 0.957 |
| **VN-1** | 0.861 |  | 1.000 | 0.986 | 1.000 | 0.933 | 0.933 | 0.933 | 0.933 |
| **VN-2** | 0.860 | 0.998 |  | 0.986 | 1.000 | 0.933 | 0.933 | 0.933 | 0.933 |
| **VN-6** | 0.857 | 0.985 | 0.985 |  | 0.986 | 0.927 | 0.927 | 0.927 | 0.927 |
| **VN-7** | 0.860 | 0.998 | 0.997 | 0.984 |  | 0.933 | 0.933 | 0.933 | 0.933 |
| **VN-3** | 0.951 | 0.845 | 0.846 | 0.845 | 0.844 |  | 0.970 | 0.993 | 0.990 |
| **VN-5** | 0.967 | 0.859 | 0.858 | 0.856 | 0.858 | 0.966 |  | 0.963 | 0.960 |
| **VN-8** | 0.947 | 0.844 | 0.843 | 0.838 | 0.843 | 0.983 | 0.953 |  | 0.990 |
| **VN-4** | 0.949 | 0.848 | 0.847 | 0.845 | 0.847 | 0.986 | 0.957 | 0.984 |  |

^1^ Upper half shows amino acid similarity; lower half shows nucleotide similarity

**Supplementary Table S3c** Similarity (%)^1^ of the Capsid gene sequences of Vietnamese Canine circovirus (CanineCV) with the NC_020904 reference strain

|  |  | **CanineCV-3** | | | | **CanineCV-1** | | | |
| --- | --- | --- | --- | --- | --- | --- | --- | --- | --- |
|  | **NC_020904** | **VN-1** | **VN-2** | **VN-6** | **VN-7** | **VN-3** | **VN-5** | **VN-8** | **VN-4** |
| **NC_020904** |  | 0.948 | 0.959 | 0.959 | 0.955 | 0.974 | 0.970 | 0.977 | 0.974 |
| **VN-1** | 0.891 |  | 0.988 | 0.988 | 0.985 | 0.929 | 0.929 | 0.940 | 0.940 |
| **VN-2** | 0.896 | 0.991 |  | 1.000 | 0.996 | 0.940 | 0.940 | 0.951 | 0.951 |
| **VN-6** | 0.896 | 0.991 | 0.997 |  | 0.996 | 0.940 | 0.940 | 0.951 | 0.951 |
| **VN-7** | 0.895 | 0.990 | 0.998 | 0.996 |  | 0.937 | 0.937 | 0.948 | 0.948 |
| **VN-3** | 0.913 | 0.853 | 0.858 | 0.858 | 0.857 |  | 0.996 | 0.974 | 0.977 |
| **VN-5** | 0.913 | 0.858 | 0.863 | 0.863 | 0.862 | 0.990 |  | 0.970 | 0.981 |
| **VN-8** | 0.929 | 0.857 | 0.862 | 0.862 | 0.861 | 0.942 | 0.942 |  | 0.981 |
| **VN-4** | 0.926 | 0.863 | 0.868 | 0.868 | 0.867 | 0.961 | 0.969 | 0.963 |  |

^1^Upper half shows amino acid similarity; lower half shows nucleotide similarity

**Supplementary Table S4** Relevant amino acid^1^ changes in the Replicase (Rep) protein sequence of each Canine circovirus (CanineCV) genotype

| **Accession No** | **Country** | **Year** | **Host^3^** | **Genotype** | **Rep protein (aa)** | | | | | | | | | | |
| --- | --- | --- | --- | --- | --- | --- | --- | --- | --- | --- | --- | --- | --- | --- | --- |
|  |  |  |  |  | **32** | **35** | **69** | **97** | **141** | **149** | **164** | **177** | **211** | **231** | **249** |
| JQ821392 | USA | 2011 | Dog | CanineCV-1 | E | D | R | R | F | Y | T | C | Q | C | A |
| KC241982 | USA | 2011 | Dog | CanineCV-1 | E | D | R | R | Y | Y | T | C | Q | C | A |
| KC241984 | USA | 2011 | Dog | CanineCV-1 | E | D | R | R | Y | Y | T | C | Q | C | A |
| KF887949 | Germany | 2013 | Dog | CanineCV-1 | E | D | Q | R | F | Y | A | C | Q | C | A |
| KJ530972 | Italy | 2013 | Dog | CanineCV-1 | E | D | R | R | Y | Y | T | C | Q | C | A |
| KT283604 | Germany | 2014 | Dog | CanineCV-1 | E | D | Q | R | F | Y | T | C | Q | T | A |
| KT734812 | Italy | 2014 | Wolf | CanineCV-1 | E | D | R | R | Y | Y | T | C | Q | C | A |
| KT734813 | Italy | 2013 | Dog | CanineCV-1 | E | D | Q | R | F | Y | T | C | Q | C | A |
| KT734814 | Italy | 2013 | Wolf | CanineCV-1 | E | D | R | R | Y | Y | T | C | Q | C | A |
| KT734816 | Italy | 2013 | Badger | CanineCV-1 | E | D | N | R | F | Y | T | C | Q | C | A |
| KT734820 | Italy | 2013 | Wolf | CanineCV-1 | E | D | R | R | Y | Y | T | C | Q | C | A |
| KT734821 | Italy | 2013 | Dog | CanineCV-1 | E | D | K | R | F | Y | A | C | Q | C | A |
| KT734822 | Italy | 2013 | Wolf | CanineCV-1 | E | D | R | R | Y | F | T | C | Q | C | A |
| KT734823 | Italy | 2013 | Dog | CanineCV-1 | E | D | R | R | Y | Y | T | C | Q | C | A |
| KT734826 | Italy | 2013 | Dog | CanineCV-1 | E | D | R | R | Y | Y | T | C | Q | C | A |
| KT734828 | Italy | 2014 | Wolf | CanineCV-1 | E | D | R | R | Y | Y | T | C | Q | C | A |
| MF457592 | USA | 2015 | Dog | CanineCV-1 | E | D | R | R | Y | Y | T | C | Q | C | A |
| MG266899 | China | 2016 | Dog | CanineCV-1 | E | D | R | R | F | Y | T | C | Q | C | A |
| MK033608 | Argentina | 2016 | Dog | CanineCV-1 | E | D | Q | R | Y | Y | T | C | Q | C | A |
| MK424788 | Brazil | 2014 | Dog | CanineCV-1 | E | D | R | R | F | Y | T | C | Q | C | A |
| MT293519 | Colombia | 2019 | Dog | CanineCV-1 | E | D | R | R | Y | Y | T | C | Q | C | A |
| MT293520 | Colombia | 2019 | Dog | CanineCV-1 | E | D | R | R | Y | Y | T | C | Q | C | A |
| MT293521 | Colombia | 2019 | Dog | CanineCV-1 | E | D | R | R | Y | Y | T | C | Q | C | A |
| MT740198 | Vietnam^2^ | 2017 | Dog | CanineCV-1 | E | D | R | R | Y | Y | T | C | Q | C | A |
| MT740199 | Vietnam^2^ | 2017 | Dog | CanineCV-1 | E | D | R | R | Y | Y | T | C | Q | C | A |
| MT740200 | Vietnam^2^ | 2017 | Dog | CanineCV-1 | E | D | R | R | Y | Y | T | C | Q | C | A |
| MT740201 | Vietnam^2^ | 2017 | Dog | CanineCV-1 | E | D | R | R | Y | Y | T | C | Q | C | A |
| KY388483 | China | 2015 | Dog | CanineCV-2 | A | D | N | R | Y | F | T | C | Q | L | A |
| KY388484 | China | 2015 | Dog | CanineCV-2 | A | D | N | R | Y | F | A | C | Q | L | A |
| KY388485 | China | 2015 | Dog | CanineCV-2 | E | D | K | K | Y | F | T | C | Q | L | A |
| KY388486 | China | 2014 | Dog | CanineCV-2 | A | D | N | R | Y | F | T | C | Q | L | A |
| KY388487 | China | 2014 | Dog | CanineCV-2 | A | D | N | R | Y | F | T | C | Q | L | A |
| KY388488 | China | 2014 | Dog | CanineCV-2 | A | D | N | R | Y | F | T | C | Q | L | A |
| KY388497 | China | 2015 | Dog | CanineCV-2 | A | D | N | R | Y | F | A | C | Q | L | A |
| KY388498 | China | 2015 | Dog | CanineCV-2 | E | E | N | R | Y | Y | T | C | Q | L | A |
| KY388499 | China | 2015 | Dog | CanineCV-2 | A | D | N | R | Y | F | T | C | Q | L | A |
| KY388501 | China | 2015 | Dog | CanineCV-2 | A | D | N | R | Y | F | N | C | Q | L | A |
| MG279118 | China | 2017 | NA | CanineCV-2 | A | D | N | R | Y | F | T | C | Q | L | A |
| MG279121 | China | 2017 | NA | CanineCV-2 | A | D | N | R | Y | F | T | C | S | L | A |
| MG279125 | China | 2017 | NA | CanineCV-2 | A | D | N | R | Y | F | T | C | P | L | A |
| MG279132 | China | 2017 | NA | CanineCV-2 | A | D | N | R | Y | F | T | C | Q | L | A |
| MG279139 | China | 2017 | NA | CanineCV-2 | A | D | N | R | Y | F | T | C | Q | L | A |
| MG279140 | China | 2017 | NA | CanineCV-2 | A | D | N | R | Y | F | T | C | P | L | A |
| KY388494 | China | 2016 | Dog | CanineCV-3 | E | D | K | K | Y | F | T | C | Q | L | A |
| KY388495 | China | 2015 | Dog | CanineCV-3 | E | D | K | K | Y | F | T | C | Q | L | A |
| KY388496 | China | 2015 | Dog | CanineCV-3 | E | D | K | K | Y | F | T | C | Q | L | A |
| MF797786 | China | 2016 | Dog | CanineCV-3 | E | D | K | K | Y | F | T | C | Q | C | A |
| MK731981 | China | 2016 | Dog | CanineCV-3 | E | E | K | K | Y | F | T | C | Q | C | A |
| MK731982 | China | 2016 | Dog | CanineCV-3 | E | D | K | K | Y | F | T | C | Q | L | A |
| MK944079 | China | 2016 | Dog | CanineCV-3 | E | D | K | K | Y | F | T | C | Q | L | A |
| MK944080 | China | 2016 | Dog | CanineCV-3 | E | D | K | K | Y | F | T | C | Q | L | A |
| MN128702 | China | 2018 | Dog | CanineCV-3 | E | D | K | K | Y | F | T | C | Q | L | A |
| MT740194 | Vietnam^2^ | 2017 | Dog | CanineCV-3 | E | D | K | K | Y | F | T | C | Q | C | A |
| MT740195 | Vietnam^2^ | 2017 | Dog | CanineCV-3 | E | D | K | K | Y | F | T | C | Q | C | A |
| MT740196 | Vietnam^2^ | 2017 | Dog | CanineCV-3 | E | D | K | K | Y | F | T | C | Q | C | A |
| MT740197 | Vietnam^2^ | 2017 | Dog | CanineCV-3 | E | D | K | K | Y | F | T | C | Q | C | A |
| KT946839 | China | 2014 | Dog | CanineCV-4 | E | E | N | R | Y | Y | T | C | Q | L | A |
| KY388480 | China | 2015 | Dog | CanineCV-4 | E | E | N | R | F | Y | T | C | Q | L | A |
| KY388481 | China | 2014 | Dog | CanineCV-4 | E | E | N | R | Y | Y | T | C | Q | L | A |
| KY388482 | China | 2015 | Dog | CanineCV-4 | E | E | N | R | F | Y | T | C | Q | L | A |
| KY388489 | China | 2014 | Dog | CanineCV-4 | E | E | N | R | Y | Y | T | C | Q | L | A |
| KY388490 | China | 2014 | Dog | CanineCV-4 | E | E | N | R | Y | Y | T | C | Q | L | A |
| KP260925 | UK | 2013 | Fox | CanineCV-5 | E | D | K | Q | H | F | A | V | S | C | G |
| KP260926 | UK | 2013 | Fox | CanineCV-5 | E | D | K | Q | H | F | A | V | S | C | G |
| KP260927 | UK | 2013 | Fox | CanineCV-5 | E | D | K | Q | H | F | A | V | S | C | G |
| MT180077 | Norway | 2015 | Fox | CanineCV-5 | E | D | K | Q | H | F | A | V | S | C | G |
| MT180078 | Norway | 2014 | Fox | CanineCV-5 | E | D | K | Q | H | F | A | V | S | C | G |
| MT180079 | Norway | 2014 | Fox | CanineCV-5 | E | D | K | R | H | F | A | V | S | C | G |
| MT180080 | Norway | 2017 | Fox | CanineCV-5 | E | D | K | Q | H | F | A | V | S | C | G |
| MT180081 | Normal | 2017 | Fox | CanineCV-5 | E | D | K | Q | H | F | A | V | S | C | G |
| MT180082 | Normal | 2017 | Fox | CanineCV-5 | E | D | K | Q | H | F | T | V | S | C | G |
| MT180083 | Norway | 1996 | Fox | CanineCV-5 | E | D | K | R | H | F | A | V | S | C | G |
| MT180084 | Normal | 1997 | Fox | CanineCV-5 | E | D | K | R | H | F | A | V | S | C | G |
| MT180085 | Norway | 1999 | Fox | CanineCV-5 | E | D | K | R | H | F | A | V | S | C | G |
| MT180086 | Norway | 1998 | Fox | CanineCV-5 | E | D | K | R | H | F | A | V | S | C | G |
| MT180087 | Norway | 1997 | Fox | CanineCV-5 | E | D | K | R | H | F | A | V | S | C | G |
| MT180088 | Norway | 1999 | Fox | CanineCV-5 | E | D | K | R | H | F | A | V | S | C | G |
| MT180089 | Norway | 1997 | Fox | CanineCV-5 | E | D | K | R | H | F | A | V | S | C | G |
| MT180090 | Norway | 1997 | Fox | CanineCV-5 | E | D | K | R | H | F | A | V | S | C | G |

^1^ Amino acid symbol: A (Alanine), R (Arginine), N (Asparagine); D (Aspartic acid), C (Cysteine), E (Glutamic acid), Q (Glutamine), G (Glycine), H (Histidine), I (Isoleucine), L (Leucine), K (Lysine), M (Methionine), F (Phenylalanine), P (Proline), S (Serine), T (Threonine), Y (Tyrosine), and V (Valine)

^2^ Vietnamese strains from this study

^3^NA (no data available)

**Supplementary Table S5** Relevant amino acid^1^ changes in the Capsid (Cap) protein sequence of each Canine circovirus (CanineCV) genotype

| **Accession No** | **Country** | **Year** | **Host** | **Genotype** | **Cap protein (aa)** | | | | | | | | | | | | | | | |
| --- | --- | --- | --- | --- | --- | --- | --- | --- | --- | --- | --- | --- | --- | --- | --- | --- | --- | --- | --- | --- |
|  |  |  |  |  | **13** | **28** | **29** | **57** | **83** | **94** | **95** | **144** | **149** | **150** | **193** | **195** | **208** | **211** | **239** | **240** |
| JQ821392 | USA | 2011 | Dog | CanineCV-1 | N | N | R | Q | T | F | Y | T | L | E | D | T | T | V | P | D |
| KC241982 | USA | 2011 | Dog | CanineCV-1 | S | N | R | Q | T | F | Y | T | L | E | D | A | T | V | P | D |
| KC241984 | USA | 2011 | Dog | CanineCV-1 | S | N | R | Q | T | F | Y | T | L | E | D | A | T | V | P | D |
| KF887949 | Germany | 2013 | Dog | CanineCV-1 | S | N | R | Q | I | Y | Y | T | L | E | D | T | T | V | P | D |
| KJ530972 | Italy | 2013 | Dog | CanineCV-1 | S | N | R | Q | T | F | Y | T | L | E | D | T | T | V | P | D |
| KT283604 | Germany | 2014 | Dog | CanineCV-1 | S | N | R | Q | I | F | Y | S | L | E | D | T | T | V | P | D |
| KT734812 | Italy | 2014 | Wolf | CanineCV-1 | S | N | R | Q | T | F | Y | T | L | E | D | T | T | V | P | D |
| KT734813 | Italy | 2013 | Dog | CanineCV-1 | S | N | R | Q | T | F | Y | T | L | E | D | T | T | V | P | D |
| KT734814 | Italy | 2013 | Wolf | CanineCV-1 | S | N | R | Q | T | F | Y | T | L | E | D | T | T | V | P | D |
| KT734816 | Italy | 2013 | Badger | CanineCV-1 | S | N | R | Q | T | F | Y | T | L | E | D | T | T | V | P | D |
| KT734820 | Italy | 2013 | Wolf | CanineCV-1 | S | N | R | Q | T | F | Y | T | L | E | D | T | T | V | P | D |
| KT734821 | Italy | 2013 | Dog | CanineCV-1 | S | N | R | Q | T | F | Y | T | L | E | D | T | T | V | P | D |
| KT734822 | Italy | 2013 | Wolf | CanineCV-1 | S | N | R | Q | I | F | Y | T | L | E | N | T | T | V | P | D |
| KT734823 | Italy | 2013 | Dog | CanineCV-1 | S | N | R | Q | T | F | Y | T | L | E | D | T | T | V | P | D |
| KT734826 | Italy | 2013 | Dog | CanineCV-1 | S | N | R | Q | T | F | Y | T | L | E | D | T | T | V | P | D |
| KT734828 | Italy | 2014 | Wolf | CanineCV-1 | S | N | R | Q | T | F | Y | T | L | E | D | T | T | V | P | D |
| MF457592 | USA | 2015 | Dog | CanineCV-1 | S | N | R | Q | T | F | Y | T | L | E | D | T | T | V | P | D |
| MG266899 | China | 2016 | Dog | CanineCV-1 | S | N | R | Q | T | F | Y | T | L | E | D | T | T | I | P | D |
| MK033608 | Argentina | 2016 | Dog | CanineCV-1 | S | N | R | Q | T | F | F | C | L | E | D | T | T | V | P | D |
| MK424788 | Brazil | 2014 | Dog | CanineCV-1 | S | N | R | Q | T | F | Y | T | L | E | D | T | T | V | P | D |
| MT293519 | Colombia | 2019 | Dog | CanineCV-1 | S | N | R | Q | T | F | Y | T | L | E | D | T | T | V | P | D |
| MT293520 | Colombia | 2019 | Dog | CanineCV-1 | S | N | R | Q | T | F | Y | T | L | E | D | T | T | V | P | D |
| MT293521 | Colombia | 2019 | Dog | CanineCV-1 | S | N | R | Q | T | F | Y | T | L | E | D | T | T | V | P | D |
| MT740198 | Vietnam^2^ | 2017 | Dog | CanineCV-1 | S | N | R | Q | T | F | Y | T | L | E | D | T | T | I | P | D |
| MT740199 | Vietnam^2^ | 2017 | Dog | CanineCV-1 | S | N | R | Q | T | F | Y | T | L | E | D | T | T | I | P | D |
| MT740200 | Vietnam^2^ | 2017 | Dog | CanineCV-1 | S | N | R | Q | T | F | Y | T | L | E | D | T | T | V | P | D |
| MT740201 | Vietnam^2^ | 2017 | Dog | CanineCV-1 | S | N | R | Q | T | F | Y | T | L | E | D | T | T | I | P | D |
| KY388483 | China | 2015 | Dog | CanineCV-2 | R | N | N | T | T | Y | F | T | N | Q | E | Q | T | I | A | E |
| KY388484 | China | 2015 | Dog | CanineCV-2 | R | N | N | T | T | Y | F | T | S | Q | E | Q | N | I | A | E |
| KY388485 | China | 2015 | Dog | CanineCV-2 | R | N | N | T | T | Y | F | T | S | Q | E | Q | N | I | A | E |
| KY388486 | China | 2014 | Dog | CanineCV-2 | R | N | N | T | T | Y | F | S | S | Q | E | Q | N | I | A | E |
| KY388487 | China | 2014 | Dog | CanineCV-2 | R | N | N | T | T | Y | F | T | N | Q | E | Q | N | I | A | E |
| KY388488 | China | 2014 | Dog | CanineCV-2 | R | N | N | Q | T | Y | F | T | S | Q | E | Q | N | I | A | E |
| KY388497 | China | 2015 | Dog | CanineCV-2 | R | N | N | T | T | Y | F | T | N | Q | E | Q | T | I | A | E |
| KY388498 | China | 2015 | Dog | CanineCV-2 | R | N | N | T | T | Y | F | T | S | Q | E | Q | N | I | A | E |
| KY388499 | China | 2015 | Dog | CanineCV-2 | R | N | N | T | T | Y | F | T | N | Q | E | Q | T | I | A | E |
| KY388501 | China | 2015 | Dog | CanineCV-2 | R | N | N | T | T | Y | F | T | S | Q | E | Q | N | I | A | E |
| MG279118 | China | 2017 | NA | CanineCV-2 | R | N | N | T | T | Y | F | T | S | Q | E | Q | T | I | A | E |
| MG279121 | China | 2017 | NA | CanineCV-2 | R | N | N | T | T | Y | F | S | S | Q | E | Q | N | I | A | E |
| MG279125 | China | 2017 | NA | CanineCV-2 | R | N | N | T | T | Y | F | S | S | Q | E | Q | N | I | A | E |
| MG279132 | China | 2017 | NA | CanineCV-2 | R | N | N | T | T | Y | F | S | S | Q | E | Q | N | I | A | E |
| MG279139 | China | 2017 | NA | CanineCV-2 | R | N | N | T | T | Y | F | S | S | Q | E | Q | N | I | A | E |
| MG279140 | China | 2017 | NA | CanineCV-2 | R | N | N | T | T | Y | F | S | S | Q | E | Q | N | I | A | E |
| KY388494 | China | 2016 | Dog | CanineCV-3 | R | N | N | Q | T | F | Y | T | G | E | D | T | T | V | P | E |
| KY388495 | China | 2015 | Dog | CanineCV-3 | R | N | N | Q | T | F | F | T | H | E | D | T | T | V | P | E |
| KY388496 | China | 2015 | Dog | CanineCV-3 | R | N | N | Q | T | F | F | T | H | E | D | T | T | V | P | E |
| MF797786 | China | 2016 | Dog | CanineCV-3 | S | N | N | Q | T | F | Y | T | S | E | D | T | T | V | P | E |
| MK731981 | China | 2016 | Dog | CanineCV-3 | R | N | N | Q | T | F | Y | T | G | E | D | T | T | V | P | E |
| MK731982 | China | 2016 | Dog | CanineCV-3 | R | N | N | Q | T | F | Y | T | G | E | D | T | T | V | P | E |
| MK944079 | China | 2016 | Dog | CanineCV-3 | S | N | N | Q | T | Y | Y | T | G | E | D | T | T | V | P | E |
| MK944080 | China | 2016 | Dog | CanineCV-3 | R | N | N | Q | T | F | Y | T | G | E | D | T | T | V | P | E |
| MN128702 | China | 2018 | Dog | CanineCV-3 | R | N | T | Q | T | F | Y | T | G | E | D | T | T | V | P | E |
| MT740194 | Vietnam^2^ | 2017 | Dog | CanineCV-3 | R | N | N | Q | A | F | H | T | G | E | D | T | T | V | P | E |
| MT740195 | Vietnam^2^ | 2017 | Dog | CanineCV-3 | R | N | N | Q | T | F | Y | T | G | E | D | T | T | V | P | E |
| MT740196 | Vietnam^2^ | 2017 | Dog | CanineCV-3 | R | N | N | Q | T | F | Y | T | G | E | D | T | T | V | P | E |
| MT740197 | Vietnam^2^ | 2017 | Dog | CanineCV-3 | R | N | N | Q | T | F | Y | T | G | E | D | T | T | V | P | E |
| KT946839 | China | 2014 | Dog | CanineCV-4 | R | N | N | Q | T | Y | Y | T | M | E | D | T | T | I | P | D |
| KY388480 | China | 2015 | Dog | CanineCV-4 | R | N | N | Q | T | F | Y | T | M | E | D | T | T | I | P | D |
| KY388481 | China | 2014 | Dog | CanineCV-4 | R | N | N | Q | T | Y | Y | T | M | E | D | T | T | I | P | D |
| KY388482 | China | 2015 | Dog | CanineCV-4 | R | N | N | Q | T | Y | Y | T | M | E | D | T | T | I | P | D |
| KY388489 | China | 2014 | Dog | CanineCV-4 | R | N | N | Q | T | Y | Y | T | M | E | D | T | T | I | P | D |
| KY388490 | China | 2014 | Dog | CanineCV-4 | R | N | N | Q | T | Y | Y | T | M | E | D | T | T | I | P | D |
| KP260925 | UK | 2013 | Fox | CanineCV-5 | R | R | N | Q | V | Y | F | A | T | E | E | S | Q | V | P | E |
| KP260926 | UK | 2013 | Fox | CanineCV-5 | R | R | N | Q | V | Y | F | A | T | E | E | S | Q | V | P | E |
| KP260927 | UK | 2013 | Fox | CanineCV-5 | R | R | N | Q | V | Y | F | A | T | E | D | S | Q | V | P | E |
| MT180077 | Norway | 2015 | Fox | CanineCV-5 | R | R | N | Q | V | Y | F | A | T | E | E | T | Q | V | P | D |
| MT180078 | Norway | 2014 | Fox | CanineCV-5 | R | R | N | R | V | Y | F | T | N | E | E | A | Q | V | S | E |
| MT180079 | Norway | 2014 | Fox | CanineCV-5 | R | R | N | Q | V | Y | F | A | T | E | E | A | Q | V | P | E |
| MT180080 | Norway | 2017 | Fox | CanineCV-5 | R | R | N | Q | V | Y | F | A | K | E | E | A | Q | V | P | E |
| MT180081 | Normal | 2017 | Fox | CanineCV-5 | R | R | N | Q | V | Y | F | A | T | E | E | A | Q | V | P | D |
| MT180082 | Normal | 2017 | Fox | CanineCV-5 | R | R | N | Q | V | Y | F | A | T | D | E | A | Q | V | L | E |
| MT180083 | Norway | 1996 | Fox | CanineCV-5 | R | R | N | Q | V | Y | Y | A | T | E | E | V | Q | V | P | E |
| MT180084 | Normal | 1997 | Fox | CanineCV-5 | R | R | N | Q | V | Y | Y | A | T | E | E | V | Q | V | P | E |
| MT180085 | Norway | 1999 | Fox | CanineCV-5 | R | R | N | Q | V | Y | Y | A | T | E | E | A | Q | V | P | E |
| MT180086 | Norway | 1998 | Fox | CanineCV-5 | R | R | N | Q | V | Y | Y | A | T | E | E | V | Q | V | P | E |
| MT180087 | Norway | 1997 | Fox | CanineCV-5 | R | R | N | Q | V | Y | Y | A | T | E | E | V | Q | V | P | E |
| MT180088 | Norway | 1999 | Fox | CanineCV-5 | R | R | N | Q | V | Y | Y | A | T | E | E | V | Q | V | P | E |
| MT180089 | Norway | 1997 | Fox | CanineCV-5 | R | R | N | Q | V | Y | Y | A | T | E | E | V | Q | V | P | E |
| MT180090 | Norway | 1997 | Fox | CanineCV-5 | R | R | N | Q | V | Y | Y | A | T | E | E | V | Q | V | P | E |

^1^ Amino acid symbol: A (Alanine), R (Arginine), N (Asparagine); D (Aspartic acid), C (Cysteine), E (Glutamic acid), Q (Glutamine), G (Glycine), H (Histidine), I (Isoleucine), L (Leucine), K (Lysine), M (Methionine), F (Phenylalanine), P (Proline), S (Serine), T (Threonine), Y (Tyrosine), and V (Valine)

^2^ Vietnamese strains from this study

^3^NA (no data available)
